# Integrating physical experiments with computational fluid dynamics to transform mosque minarets into efficient solar chimneys

**DOI:** 10.1038/s41598-024-59589-0

**Published:** 2024-04-27

**Authors:** Hesham H. Awad, Mahmoud Desouki

**Affiliations:** 1 Department of Architectural Engineering, Faculty of Engineering, Menoufeia University, Shebeen El-Kom, Egypt; 2 Department of Architectural Engineering, Nile Higher Institute for Engineering and Technology, Mansoura, Egypt

**Keywords:** Mosque minaret, Solar chimney, Passive ventilation, Computational simulation, CFD, Natural ventilation, Sustainability, Energy harvesting, Climate sciences, Environmental sciences

## Abstract

This study explores the potential of repurposing mosque minarets as solar chimneys in hot arid regions to facilitate natural ventilation and diminish the reliance on energy-intensive cooling systems. Originating as a means to call the faithful to prayer, minarets have become iconic landmarks within Islamic cities. This research focuses on Cairo, Egypt, as a representative hot arid environment. The paper traces the evolution of the minaret, underscoring the variations in form that influence the experimental design. The investigation proceeded in two stages: the construction of physical mosque models with variably positioned minarets for laboratory testing, ensuring standardized measurements, followed by computational fluid dynamics (CFD) simulations for comparison. Findings indicate that mosque minarets can be effectively adapted for passive ventilation, with their performance significantly influenced by orientation and placement. This study concludes that traditional mosque minarets offer a viable, sustainable option for passive cooling in hot climates.

## Introduction

Minarets are tall structures that are built alongside mosques. The word minaret is derived from the Arabic "Manara" which means "place of light". Historically, minarets were used to light the way for travelers at night and to call the faithful to prayer during the day. It was always used to highlight the presence of a mosque and to guide travelers as a landmark. They are often made of different construction materials, and stand tall and slender^1^.

The minaret has a rich history that dates back to the early days of Islam. There is a historical debate regarding the first minaret. Some believe it was during the era of Caliph Othman bin Affan, the third Rightly Guided Caliph^2^, while others attribute it to Ziyad bin Abih, the Umayyad Governor of Basra in 45 AHS^3^. Alternatively, al-Maqrizi credits the silos of the Amr bin al-Aas mosque, constructed by Muslim bin Mukhlad, the Governor of Egypt in 53 AH^4^. Another view holds that the first minaret was built as an additional component of the great mosque in Damascus during the seventh century by the Walid I the Umayyad Caliph. It was a simple structure that consisted of a platform and a small roof^5^. However, as Islam spread throughout the world, the minaret began to take on new forms and functions. The minaret's design and style varied depending on the region and period, some minarets being tall and slender, while others were shorter and wider. Some had elaborate decorations, while others were simple and plain. The evolution of minarets showcases a diversity in design form. In the Umayyad era, minarets were tower-like structures with square plans. This evolved during the Abbasid era into round minarets, introducing new designs such as the twisted minaret in Samarra. An example from this period is the multi-layered Ibn Tulun Mosque’s minaret in Cairo, featuring a square base, cylindrical middle, and an eight-sided top. The Fatimid era saw the emergence of tall, slender minarets ending in bulbous domes, like those of Al-Azhar Mosque in Cairo^6^. In the Mamluk era, the minarets were high and often built on balconies, lacking a distinctive base, as seen in the Sultan Hassan Mosque. The Mughal era minarets are characterized by significant height and diameter, adorned with multiple plates and plant decorations, notable in the Quwwat al-Islam Mosque in Delhi and the Taj Mahal. In the Far East, Timurid minarets in Uzbekistan were known for their barrel shapes, while the Safavid era in Iran introduced tall, circular minarets with a distinctive inclination. The Seljuk state, stretching from Central Asia to Turkey, featured various types of minarets, including polygonal shapes with ribbing and usually a single balcony^7^. Lastly, the Ottoman Empire is renowned for its slender minarets with pencil-like ends, as exemplified by the Mohammed Ali Mosque in Cairo^8^. This diversity in size and form is influenced by the parallels drawn between the minaret’s architecture and Muslim head coverings. Studies suggest that minaret design may have been inspired by contemporary fashion. This is particularly evident in the resemblance between headscarves and the tops of minarets, symbolizing the minaret as a distinctive emblem of the Islamic state across different periods^9^.

The minaret's functions have also evolved beyond its original purpose. In some regions, the minaret is used as a watchtower to keep a lookout for potential threats^10^, while in others, it is used as a symbol of the mosque’s prestige and influence in the community^11,12^. Rather it goes beyond that to be a symbol of the Islamic presence and raise controversy about its presence in different communities^13,14^. It is also used to observe the lunar phases and Inspection of the Hijri Month's Crescent. The minaret was used as an astronomical observatory^15^. A copper crescent is erected on the top of the minaret. The rounded opening of the crescent faces the direction of the qibla to direct the prayers in the surrounding streets^16^. Nowadays the muezzin recites the call to prayer with loudspeakers installed at the top of the minaret to broadcast the call across the neighborhood. But actually, the loudspeakers could be hung on any high building it could also be connected wirelessly. Prayer times can be accurately determined using astronomical tables, allowing the muezzin to simply consult a watch. This raises the question of whether a minaret is still necessary, and if so, what its function should be^4,17^. Muslims still adhere to mosques minarets a symbol of Islamic identity, reminding the community of their faith and the call to prayer^18^. The minaret evolution was always influenced by the community ever changing needs^19^. Recently the global shift towards sustainability and green architecture. That has prompted the question of whether the minaret can offer more than just its current functions. Although the primary purpose of minarets in mosques has diminished over time, and although it is an expensive element in a mosque construction, they remain an indispensable aspect due to the emotional connection in worshippers’ minds. That created the need to find a sustainable usage of the minaret. Its unique design as a tall, narrow structure, makes it a potential candidate for incorporating sustainable features such as solar chimney.

**Figure 1:**
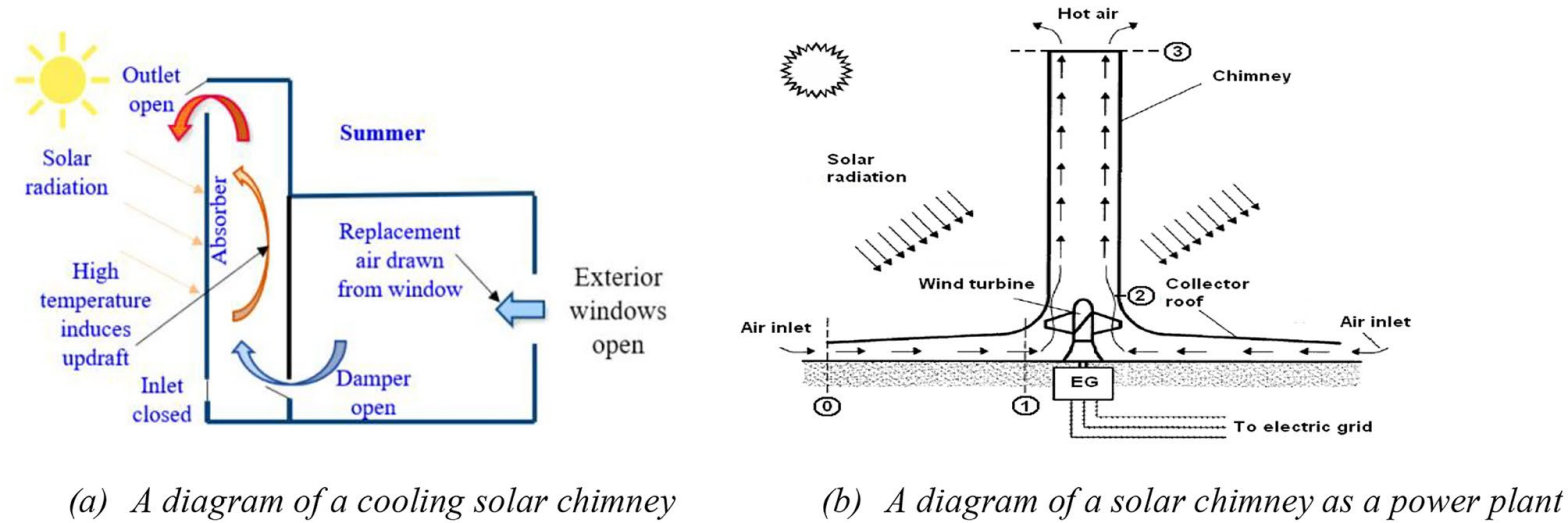
The concept of solar chimney.

A solar chimney is a passive ventilation system that uses the natural convection of air to ventilate a building. It consists of a vertical shaft, typically made of opaque glass, which is placed on the roof of a building and connected to an air intake at the base. The chimney works by allowing sunlight to heat the air inside the shaft, causing it to rise and create an updraft. This updraft then pulls in fresh air from outside and pushes out stale air from inside the building^20^. Figure 1a shows the concept. They are an innovative and sustainable solution for ventilation and passive cooling in buildings. This technology has been used for centuries in vernacular architecture, especially in hot and arid climates^21–23^. Solar chimney technology, used in power plants since the 1980s, leverages the sun's thermal energy to generate electricity through a pressure differential caused by thermal variation^24^. As shown in Fig. 1b, Recently, with the increasing concern over global warming and energy efficiency, solar chimneys have regained attention as a potential solution to reduce energy consumption in buildings^25^. Solar chimneys can be an effective way to improve indoor air quality and reduce energy consumption in buildings^26^. Studies have found that an integrated system with solar chimneys can reduce energy consumption by up to 50%^27^. Additionally, they can be used as part of a hybrid ventilation system, combining natural and mechanical ventilation for optimal performance^9^. Solar chimneys have also been found to be effective at reducing indoor temperatures during hot summer months^28^. This is due to their ability to draw in cooler outside air and exhaust hot indoor air, thus creating a cooling effect inside the building. Additionally, they can be used as part of an integrated design approach that combines passive cooling strategies with active cooling systems such as evaporative cooling^29^. Multi-channel solar chimney groups could be used in tunnels for natural ventilation and smoke exhaustion under various conditions^30^.

Overall, solar chimneys offer many benefits for buildings in terms of energy efficiency and indoor comfort. However, there are still some challenges associated with their implementation. For example, they require careful design considerations such as orientation and sizing to maximize their effectiveness^31^. Solar chimneys may not always be the best solution for all climates due to their reliance on sunlight for operation making them particularly efficient in hot desert regions where periods of solar radiation are prolonged^32^. In conclusion, solar chimneys offer many benefits for buildings in terms of energy efficiency and indoor comfort. However, careful consideration must be given when designing them to ensure optimal performance.

The paper aims to fill a research gap by exploring the potential of repurposing mosque minarets as solar chimneys in the hot arid regions. While the use of solar chimneys has been studied in building design, the potential of repurposing architectural elements, such as minarets, has not been explored in depth. It was explored by different methods in different contexts unlike the current study. Table 1 shows the previous literature main findings and its relationship to the current study. This research is novel in its approach of tracing the minaret form throughout Islamic history to know the design principles that influence the experiment, integrating computational modeling and physical experiments to evaluate the effectiveness of the minaret and the best variables. Replicating the physical measurements in different laboratories sets, and in computational modeling. The study objective is to investigate the feasibility of repurposing minarets as sustainable solar chimneys in hot arid regions. The results are important for promoting sustainable building practices and reducing energy consumption. Architects and building engineers could benefits from those findings. The findings can also inform the retrofitting of existing buildings with sustainable features. Ultimately, this research contributes to the broader goal of creating a more sustainable built environment.

**Table 1:** Reviewed literature.

Paper title	Main findings	Relevance	Reference
A comprehensive review on thermal performance and envelope thermal design of mosque buildings	Reviews thermal performance and envelope design in mosques, stressing the need for optimal design to improve thermal comfort and energy efficiency	It suggests to investigate how minarets, affect the thermal comfort and energy consumption, considering their design. It suggests future studying minarets as a wind catcher or cooling towers	^17^
The Minaret: A reassessment of architectural function and religious value, in light of modern technology	The minarets are no longer needed for their original purpose. It suggests reallocating funds spent on minarets towards economical alternatives	This shift represents a call to rethink mosque architecture in response to technological advancements and economic considerations	^4^
Elements of Islamic architectural heritage: Minaret	Highlights the evolving of minarets. It underscores the potential for contemporary technology and construction methods to reinvent minarets not just as symbols, but as integral, functional elements	The study suggests minarets can serve innovative roles, such as air traps, power generators, and sources of natural lighting, thus contributing to green objectives	^47^
Ventilation in a mosque—an additional purpose the minarets may serve	Explores how minarets can serve in ventilation in mosques, particularly in the warm-humid climate of dhaka. It proposes the innovative use as ventilation towers. Using coiled pipes to heat the shaft as a solar chimney	The passage highlights the significant potential for future research to delve into the specific benefits of using minarets for ventilation, focusing on the varying effects under different conditions. It also pointed to the importance of CFD software to simulate different variables	^48^
A new design of the minaret as a two-sides wind catcher integrated with the wing wall for passive evaporative cooling in hot climates	Introduces a design for a minaret acting as a two-sided wind catcher integrated with a wing wall, aimed at enhancing passive cooling in hot climates	The methodology combines computational fluid dynamics (CFD) simulations with wind tunnel experiments to evaluate the design's effectiveness	^49^
Viability of wind towers in achieving summer comfort in the hot arid regions of the Middle east	Both conventional and modern designs of wind towers can effectively provide thermal comfort in hot arid regions of the Middle East	It discusses the advantages of integrating such systems into modern building designs	^50^
The application of traditional architecture as passive design strategies for modern architecture in hot dry climate	The effectiveness of integrating traditional elements into building designs in hot dry climates, like Cairo, to improve energy efficiency	The methodology includes computer simulations analyzing building performance in terms of solar radiation, wind direction, and heating/cooling loads. Future research could explore other passive design strategies to further reduce energy consumption	^51^
A review for the applications of solar chimneys in buildings	Reviews the applications of solar chimneys in buildings for enhancing ventilation and reducing heat gain, emphasizing their potential to lower operational costs, and CO_2_ emissions	The need for further experimental studies to validate design strategies across different climates	^20^

## Methodology

This study employed a two-pronged approach to evaluate the viability of adapting a mosque minaret as a solar chimney in a hot arid region. Initially, the researchers conducted a literature review to identify relevant existing research. This will help to inform the development of the physical models and the selection of the experiment design variables. Various physical models of mosques with differently positioned minarets were constructed and subjected to a controlled laboratory setting to mimic the chosen regional climate, with standardized measurements taken for consistency. To ensure the accuracy and reliability of the experimental data, the study was conducted independently in two distinct laboratories using two separate devices each calibrated with identical standards. This approach facilitated the verification and validation of the measurement results.

Following the physical experiment, computational fluid dynamics (CFD) software was utilized for a computational simulation to virtually analyze the minaret's performance as a solar chimney, mirroring the setup of the physical experiment for accurate comparison. CFD software is a tool that simulates the flow of gases and liquids, heat transfer, and associated phenomena in a virtual environment. It uses numerical analysis and algorithms to solve and analyze problems involving fluid flows. This is particularly useful in situations where real-world testing might be impractical, expensive, or dangerous^33^. Researchers rely on CFD software for its user-friendly interface and automation capabilities, making it accessible to a wider range of users^34^. CFD is a powerful tool for predicting fluid behavior, its accuracy is highly dependent on the user's expertise and the specific application. CFD's accuracy in modeling fluid flows and heat transfer is highly dependent on several factors. It requires validation with experimental data^35^. A range of educational and research institutes have developed and utilized CFD software for teaching and research purposes. FlowLab, a CFD-based educational software, has been used by over 30 universities worldwide^36^. The need for cooperation between universities and academic institutions in teaching CFD modeling has been emphasized, with examples from the Moscow Aviation Institute^37^. Utilizing CFD aligns with the study objective to evaluate the effectiveness of the minaret as a solar chimney and the best variables.

**Figure 2:**
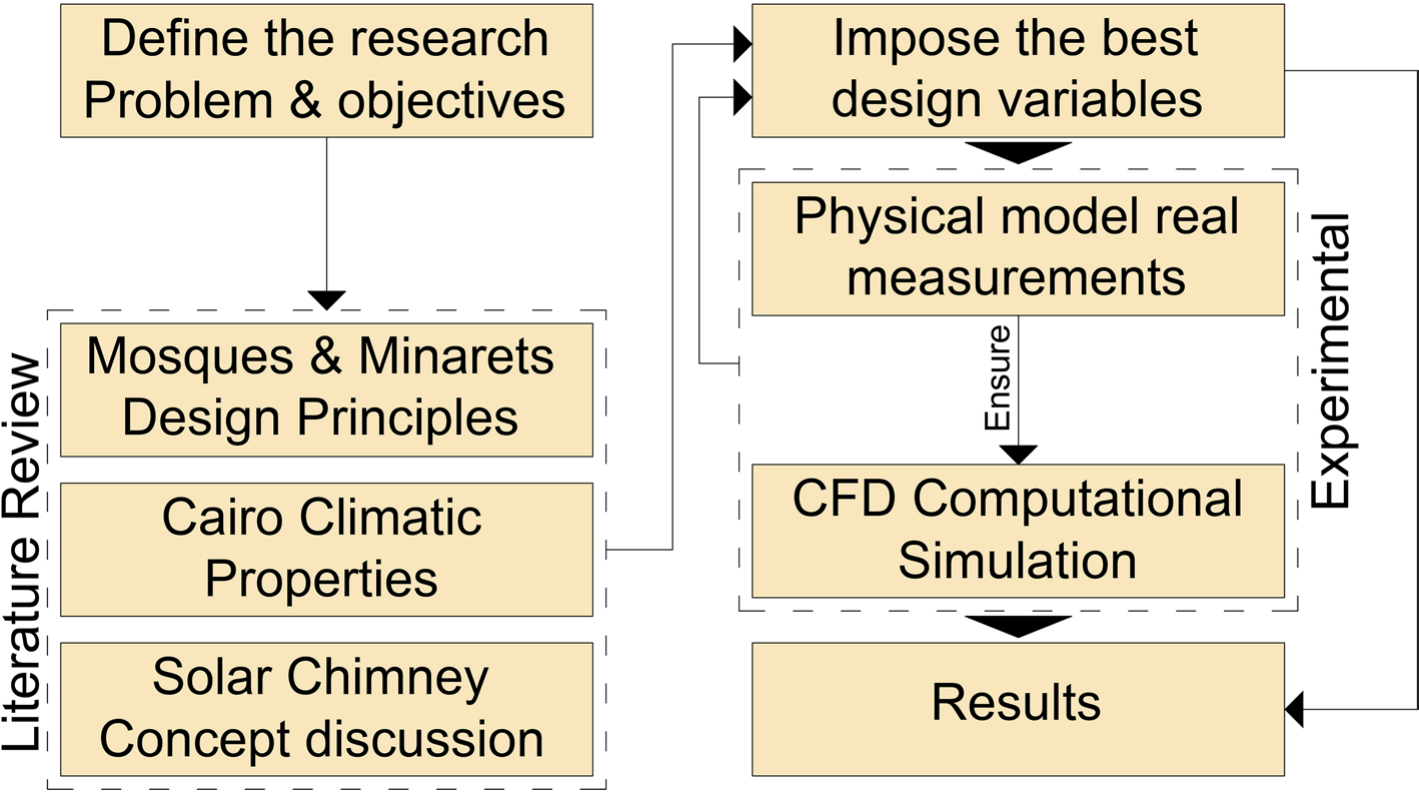
Research steps.

The juxtaposition of physical and computational results helped validate and verify the model and shed light on how the minaret's orientation and location affect the efficiency of the solar chimney mechanism. This methodology aims to provide a solid basis for transforming traditional mosque minarets into sustainable solar chimneys, contributing to the reduction of reliance on energy-intensive cooling systems in hot arid regions. Figure 2 shows the research steps.

### Imposing the experiment variables

The researchers in this study took a thorough approach in preparing for both the physical experiment and the computational simulation, ensuring that all variables relevant to the experiment were carefully considered and accurately represented. The dimensions, and proportions of the mosque and minaret were reviewed to construct the physical model. The Climatic properties were collected to mimic the regional climatic characteristics in both study phases.

### The minaret proportions

**Figure 3:**
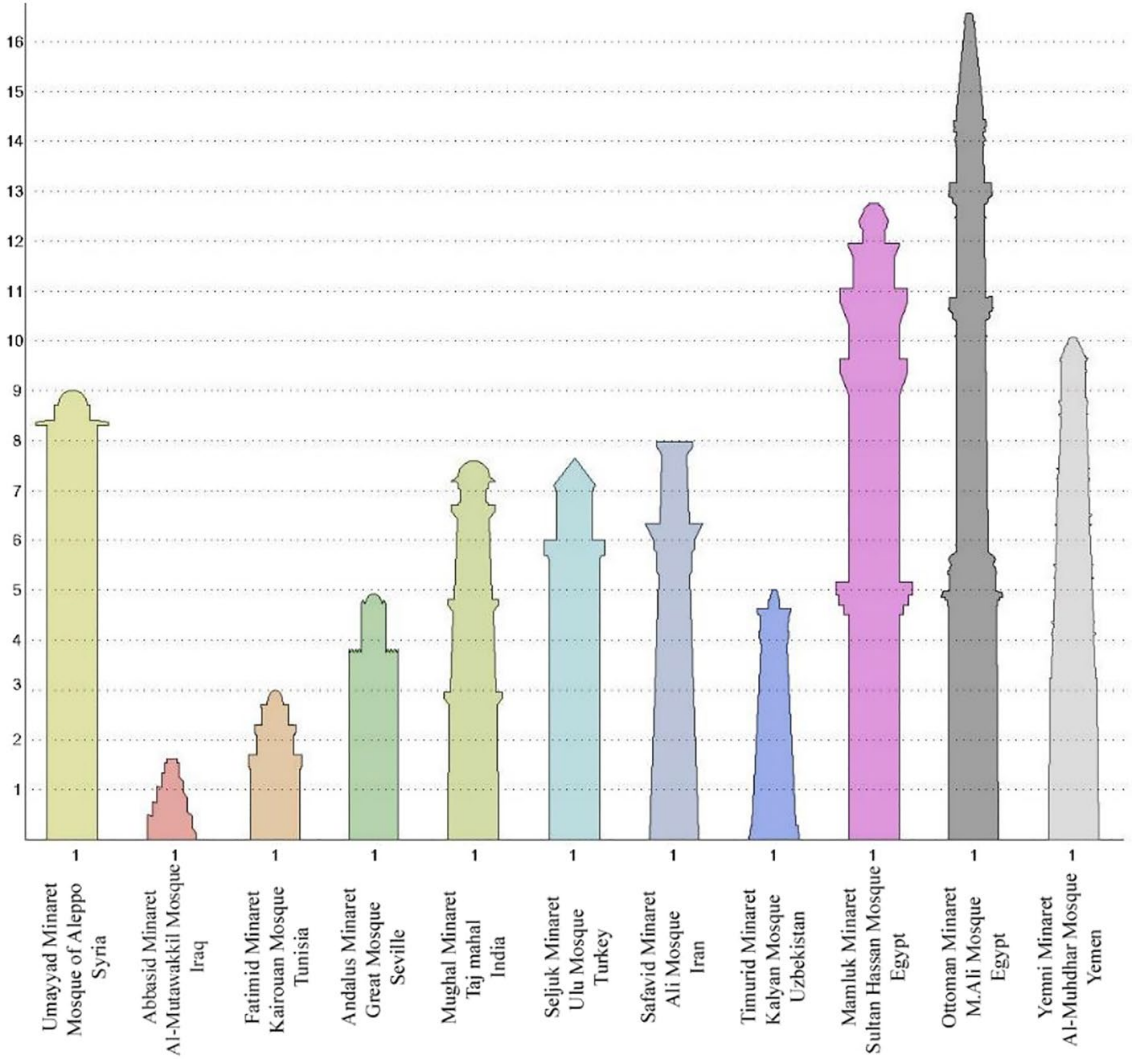
The ratio of the minaret base to its height throughout the different Islamic eras.

The proportions of minarets have varied a lot and evolved through various eras into the modern age. The researchers tracked the dimensions of a number of the most famous Islamic minarets, each of which reflects the minarets of its period and the Islamic state in which it appeared, and then deduced the approximate proportions of the different minarets, and the results were presented Fig. 3. The analysis reveals significant variations in the base-to-height ratio of minarets across Islamic history. This ratio was at its lowest, approximately 1:1.5, during the Abbasid era, and peaked in the Ottoman era, exceeding 1:16. On average, the ratio between the base and height of minarets in various Islamic periods was around 1:7 to 1:7.5. This average ratio was adopted by researchers in the experiment to maintain Islamic identity. Consequently, a square minaret was proposed with a base side length of 3.5 m and a height of 25 m.

### Cairo climatic properties

**Figure 4:**
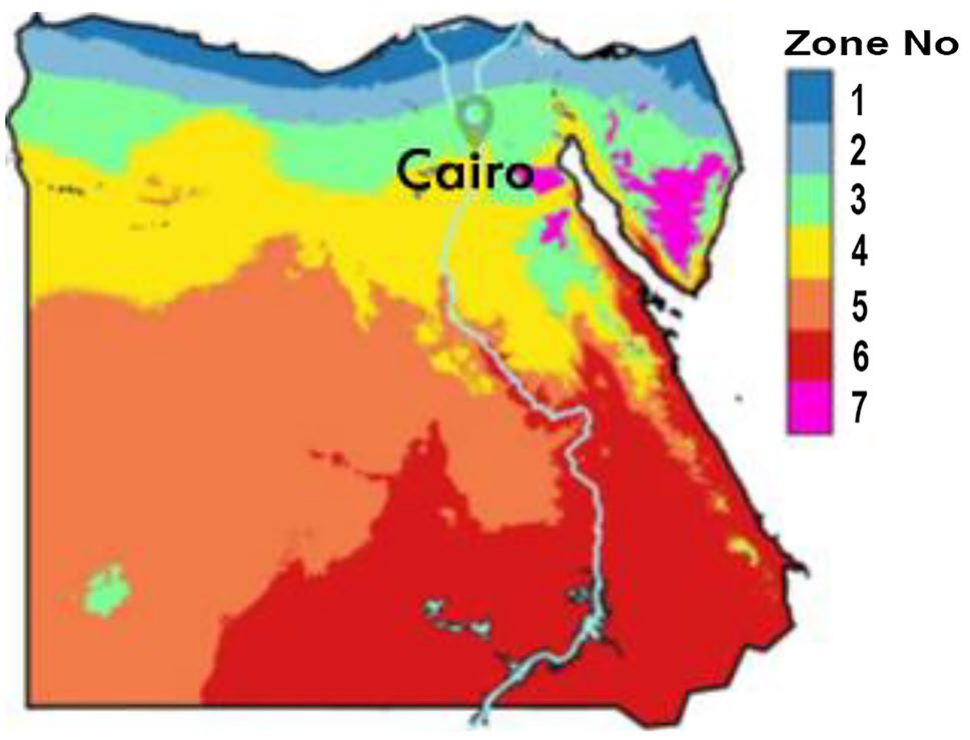
The seven climate zones in Egypt.

The study focuses on Egypt as an example of a hot, arid region, situated at the juncture of Africa and Asia within 22°–32° N latitude and 25°–35° E longitude. Which provides it with long periods of solar radiation. Egypt has a wet cool climate in winter, and a hot and dry in summer, with strong winds areas along the Red Sea and Mediterranean coasts. The average annual wind speed is 8.0–10.0 ms^−1^ along the Red Sea coast and approximately 6.0–6.5 ms^−1^ along the Mediterranean coast^38^. Recent studies, have divided Egypt into seven climatic regions for engineering purposes, as depicted in Fig. 4. Cairo, specifically, is situated in a hot, dry zone^9^. The city has a significant number of mosques, earning it the epithet 'the city of the thousand minarets’^39^. Cairo, the open museum of Islamic civilization is one of the largest Islamic cities globally, its climate data is accessible and can be summarized by the climate consultant program. That makes Cairo a perfect Candidate to experiment with. Climate Consultant is a software tool designed to assist in the creation of climate-responsive building designs. This program utilizes weather data to offer insights into the local climate. Its key features include the analysis of temperature, humidity, wind, and solar radiation, allowing architects and designers to understand the climatic conditions of a specific location comprehensively^40^.

**Figure 5:**
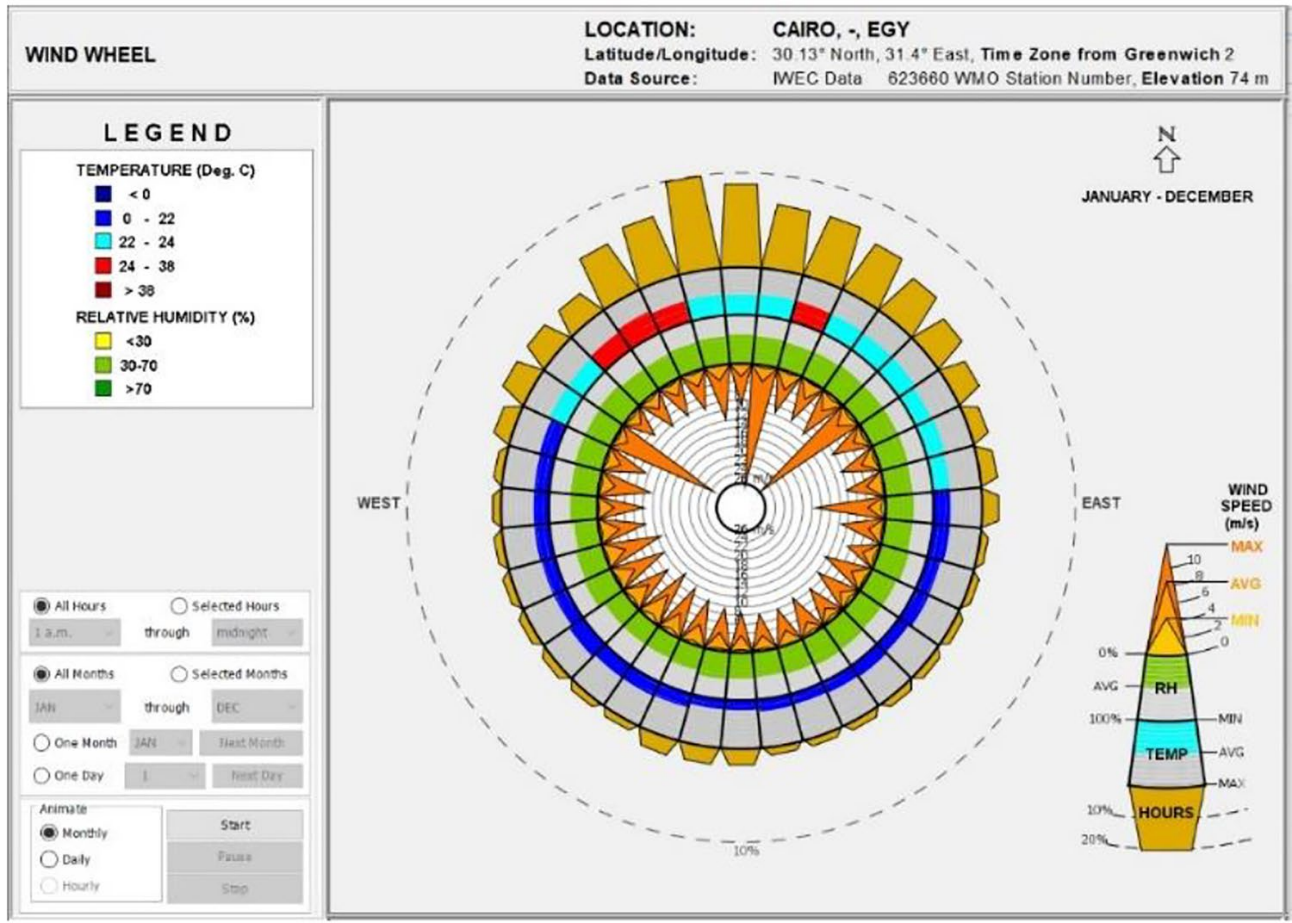
Cairo wind wheel.

The wind chart for Cairo is detailed in Fig. 5. The outermost ring indicates the percentage of wind coming from each direction, while the adjacent ring shows the average wind temperature, color-coded for different comfort levels. Another ring displays average humidity, with colors representing comfort, dry, and humid conditions. The inner circle shows wind velocities, with different shades of brown marking maximum, average, and minimum speeds. Notably, the chart excludes zero wind speed periods. It reveals that the most prolonged wind periods in Cairo are from the north and northwest, where wind temperatures are within comfortable limits and reach their peak speeds. The relative humidity of Cairo's winds predominantly falls within a 30–70% range, considered thermally comfortable.

**Figure 6:**
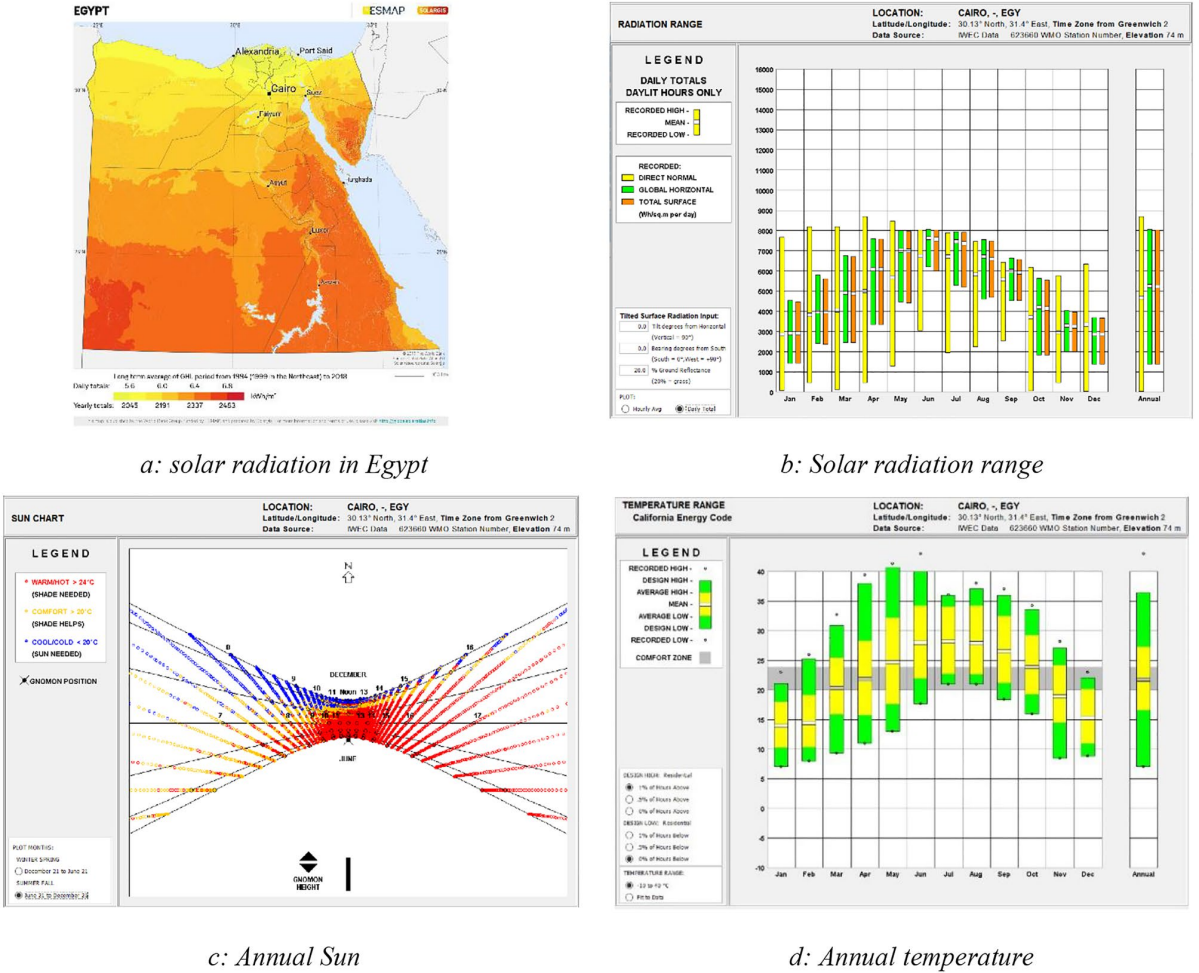
Solar radiation and temperature in Cairo.

Figure 6a shows that the Solar Atlas highlights Egypt’s status as a sunbelt country with intense direct solar radiation, varying between 2000 and 3200 kWh/m^2^/year from North to South. Sun shines daily for 9–11 h with few cloudy days annually^41^. Climate Consultant shows in Fig. 6b that the daily solar radiation average exceeds 4000 watts/m^2^ for over nine months each year, with the intensity primarily concentrated in the south in Fig. 6c. The figure also displays the shadow patterns of a gnomon at 15-min intervals throughout the year, with colored dots indicating different temperature conditions: yellow for comfort, red for overheating, and blue for underheating. These patterns suggest strategies for passive heating and cooling. Figure 6d reveals Cairo’s temperature trends, indicating that for over six months of the year, average temperatures are above the thermal comfort zone, around two months within it, and about four months below it. This data underscores the importance of considering solar radiation and temperature in architectural and urban planning in Cairo.

### Mosques dimensions

For optimal functionality design and adherence to Islamic architectural principles, Key principles included:

#### Space requirements

Rectangular plans require a net area of 1.30 m^2^ per person, while non-rectangular plans need 1.50 m^2^. The designated prayer area measures 1.20 × 0.80 m^2^ per person^42^. The overall mosque area depends on various factors, including services offered, location, and congregation size, with service areas ranging from 1.2 to 1.4 m^2^^43^.

#### Orientation and plan types

The mosque’s largest side is oriented towards the Qibla, which may require adjustments to prevent sunlight from disturbing worshippers. Preferred plan types include rectangular, square, and trapezoidal, with rectangular being the most efficient for organizing rows of worshippers and minimizing column usage. Circular or octagonal plans, which disrupt orderly rows, are discouraged.

#### Dimensions and height

Plans vary based on type and size, the height is typically one-third of its depth, ranging between 3 and 8 m for visual comfort and atmosphere^44^.

#### Entrances and windows

Entrances should accommodate at least 300 worshippers, ideally located on walls other than the Kiblah wall, preferably in the first quarter of the two side walls. The entrance should be no less than 1.5 m wide. Windows should be a minimum of 1 m high and distributed evenly for proper ventilation, with at least 20% being openable^43^.

### Physical models variables

The researchers developed a mosque model to investigate the experiment. This model, designed to hold 500 people, is typical of congregational mosques and has a rectangular shape measuring 28 × 24 m^2^ with a height of 8 m. The placement of windows and the entrance on the Kiblah facing side, located on the Eastern South wall, is to enhance worshipper convenience and comfort, as well as to align with the spiritual and functional design. The northwest wall, opposite the Kiblah, was chosen for these features due to its favorable wind direction in Cairo and its direct alignment with the Kiblah wall.

A key feature is the square-sectioned minaret, positioned on the southeastern side to optimize heat gain, a crucial consideration for the solar chimney application. The square shape choice, as opposed to a circular one, is to maximize heat gain, considering that a circular shape would result in partial shading. Additionally, the minaret’s southeastern and southwestern walls are made of dark black glass to enhance thermal capacity, and the adjacent mosque wall is insulated to prevent heat leakage. While a southwestern placement of the minaret was considered, the southeastern orientation was preferred for alignment with the northwestern wall openings. The minaret stands at a height of 25 m with a base of 3.5 m, resulting in an aspect ratio of approximately 1:7. This model encapsulates key elements, such as orientation, space allocation, and window and entrance positioning, all tailored to support the study's focus.

### Conducting the experiment

Five different scenarios for positioning the minaret as a solar chimney were tested using scaled-down mosque models (dimensions: 56 × 48 × 16 cm, representing a 1:50 scale). Each scenario had two models for thorough experimentation, resulting in a total of 18 models across nine cases. The scenarios included:

- A square-sectioned minaret is placed in the middle of the Kiblah facade, paired with a completely flat mosque roof.
- A square-sectioned minaret situated in the southwestern corner, again with a flat roof.
- Two square-sectioned minarets on the Kiblah facade, alongside a flat roof.
- A square-sectioned minaret in the middle of the Kiblah facade, with variations in the roof design—one flat roof and another featuring a shokhshekha a historical element works as lantern skylight, both with and without openings.
- A trapezoidal vertical-section minaret positioned at the center top of the mosque, complemented by a pyramidal, inclined roof.
- These first four cases were also repeated with a sloping roof and a trapezoidal minaret, making up the additional scenarios.

### Tools and devices

To ensure consistent experimental conditions, the study utilized a specially designed electric heater with a thermostat for temperature control. Additionally, an electric fan was positioned at a specific distance from each model to achieve a uniform air speed of 1 ms^−1^ at the mosque's openings (doors and windows) across all models. A wind tunnel was employed to channel air at the mosque openings, preventing any air leakage, which could impact the accuracy of the measurements. Each of the 18 models underwent two separate experiments. The first used the air source with a wind tunnel, while the second relied solely on natural air movement generated by the temperature differential inside the minaret compared to the room temperature.

**Figure 7:**
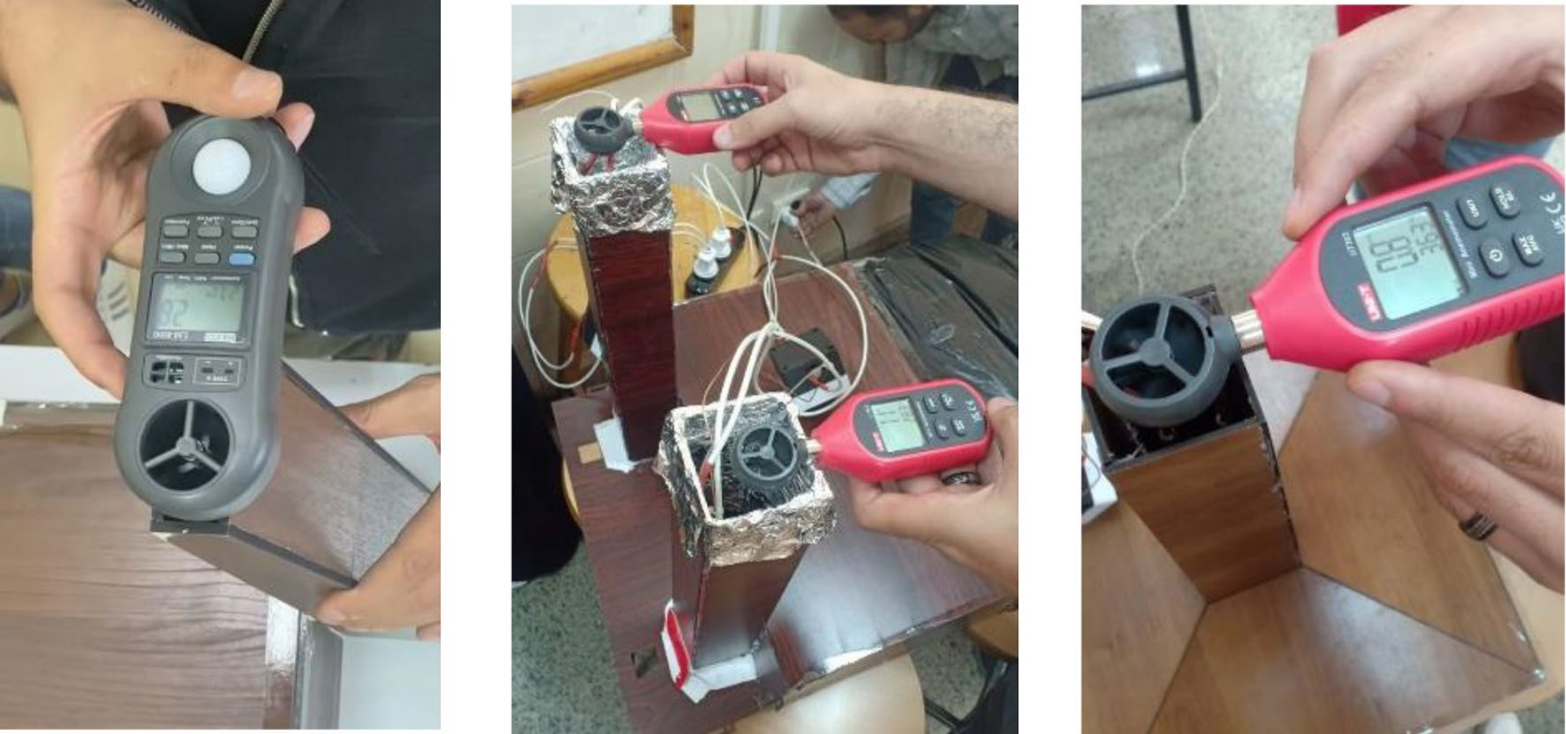
Recording various readings while conducting the experiment.

The models were constructed from medium density fiberboard to facilitate precise laser cut, ensuring accurate dimensions. To prevent air leakage, all edges and corners were sealed with a strong adhesive. Sealing all air leaks was an important challenge to get an accurate measurement. The minaret's body was insulated from the mosque’s body with a foam layer to minimize heat transfer from the minaret to the mosque. This meticulous setup aimed to provide reliable and controlled conditions for evaluating the efficiency of solar chimneys in mosques. The used measurement devices were the REED LM-8000 a compact, hand-held environmental meter that performs multiple functions like measuring air velocity, temperature, relative humidity, and light levels^45^, and the UNI-T UT363/UT363BT it is a mini anemometer, it can measure wind speed and temperature. It can measure wind speeds from 0.0 and up to 30 m per second (m/s) and it measures temperature from − 10 to 50 ℃ ^46^. Figure 7 shows taking measurements.

### Steps of the experiment

**Figure 8:**
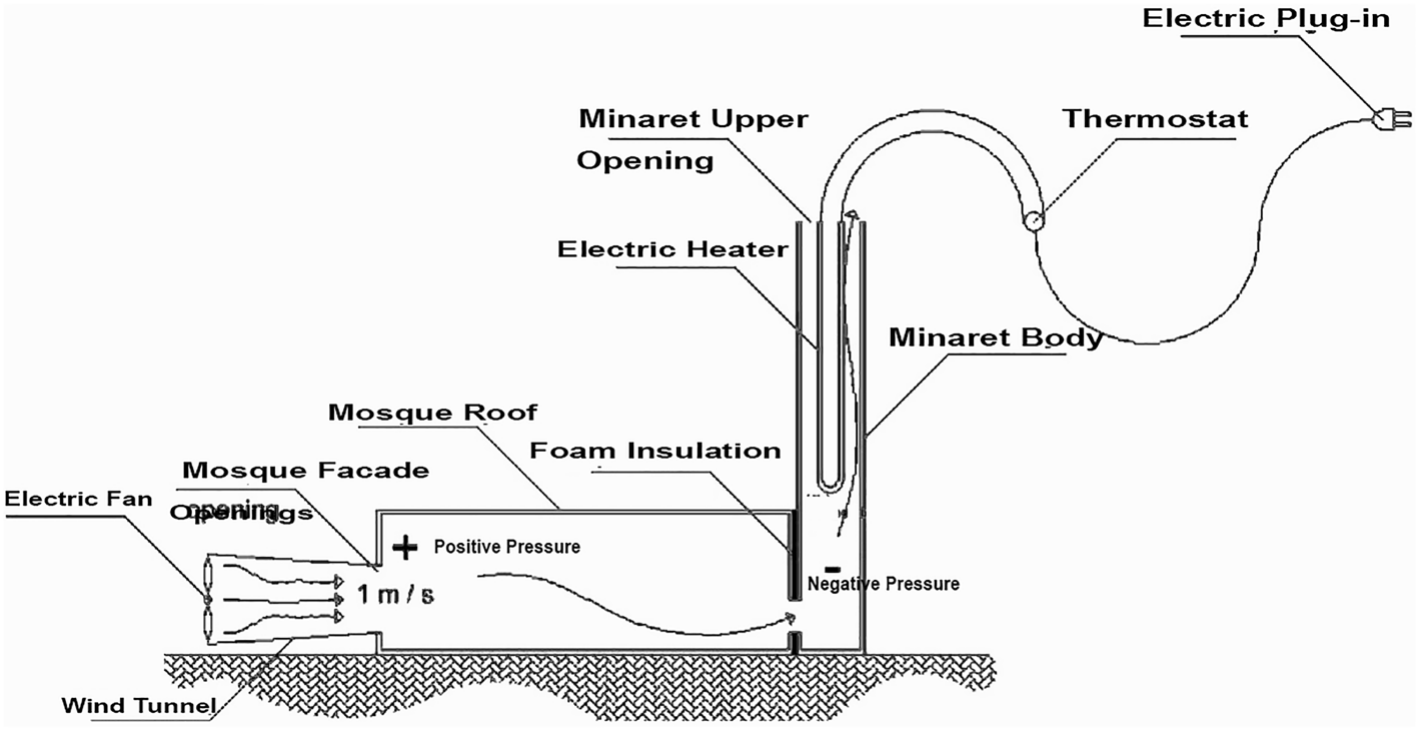
The experiment physical model section.

**Figure 9:**
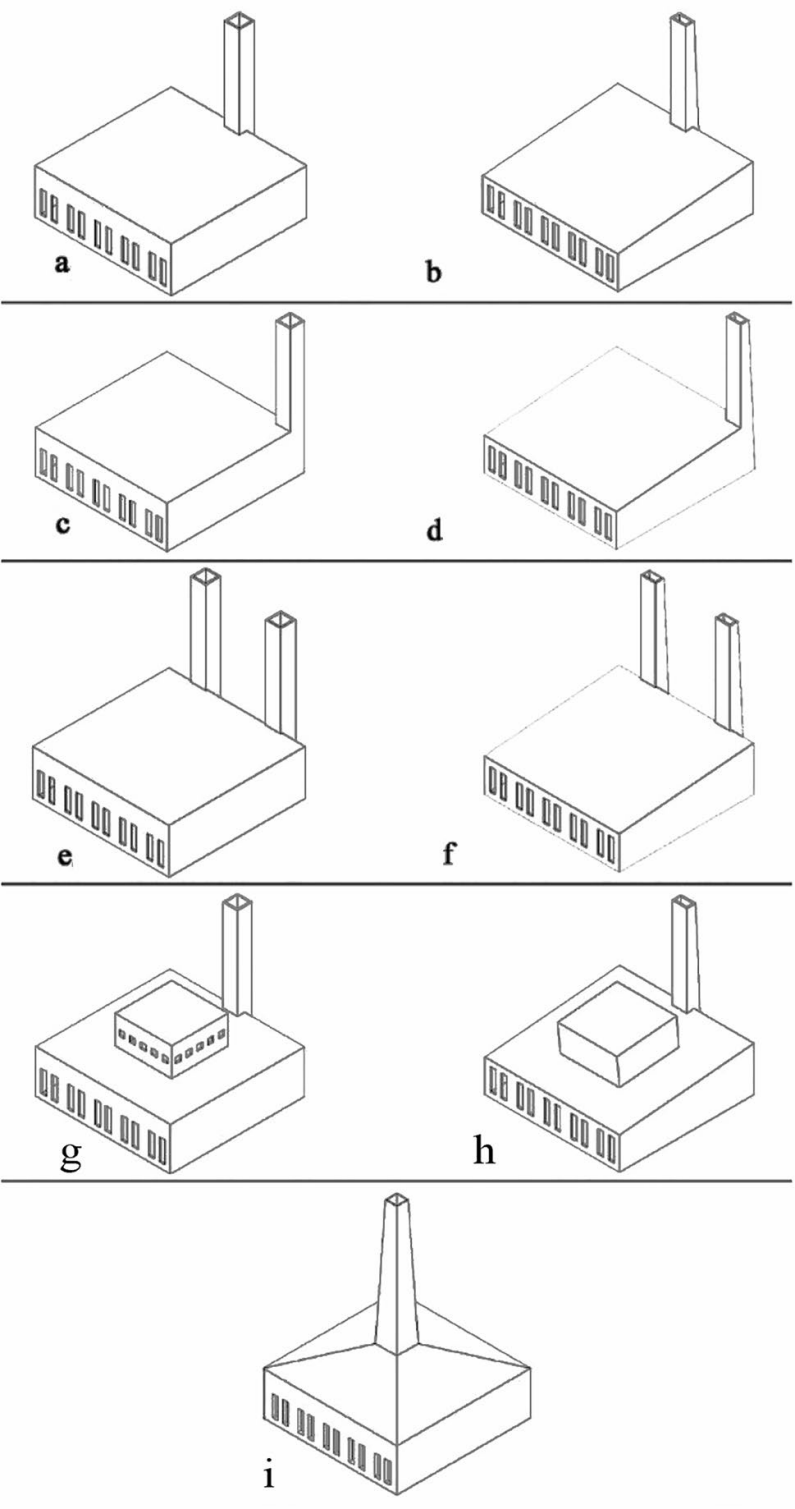
Experiment studied cases.

To conduct the experiment, the heater was set to 80° and placed inside the minaret, ensuring it did not touch the minaret's walls. The fan was then activated to generate airflow within the wind tunnel, with careful monitoring to maintain an air speed of 1 ms^−1^ at the facade openings. After a waiting period of five minutes, measurements were taken to record the air velocity and average temperature at the minaret's upper opening. This procedure was designed to assess the effectiveness of the minaret as a solar chimney under controlled conditions. Figure 8 shows a section in the physical model explaining how was the experiment conducted. Figure 9 shows the experiment different studied cases.

## Results and discussion

The results from the two laboratories sets were obtained by averaging readings from two sets of models, this approach ensured the accuracy and reliability of the findings. The findings from both laboratories showed consistency in the patterns of increase and decrease for the same model, with air speed differences ranging from 0.1 to 0.25 ms^−1^. This slight variance in speed could be attributed to differences in wind tunnel materials, affecting wind friction, or the precision of the electric heater. However, these discrepancies were deemed negligible in terms of impacting the study’s goal to identify the optimal positioning of the minaret model.

**Figure 10:**
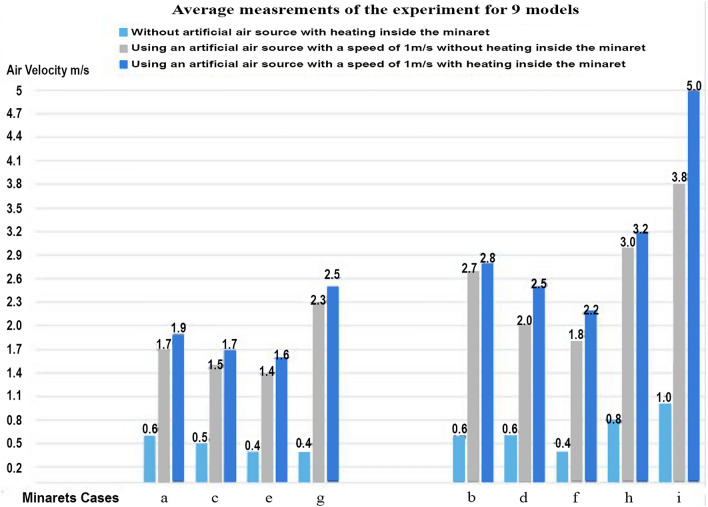
The average air speed at the upper opening of the minaret for all cases.

Table 2 and Fig. 10 present a comparative analysis of different minaret configurations and their impact on airflow and temperature within a mosque. The experimental results, detailed in Table 2, indicated several key findings regarding mosque design and natural ventilation:

1. Models with inclined roofs and sloping minarets generally outperformed those with flat roofs and regular section minarets in terms of air velocity.
2. Incorporating a shokhshekha or dome with openings didn’t enhance the minaret’s effectiveness as a solar chimney.
3. The most effective design in terms of air velocity, was similar to traditional solar chimneys used in power plants. This involved placing a sloping minaret at the center of a pyramidal or inclined roof. While structurally challenging, this design yielded optimal natural ventilation and air speed suitable for powering a turbine for energy generation.
4. Using a shokhshekha or dome without openings, particularly with an inclined roof and a sloping minaret, significantly enhanced natural ventilation and increased airspeed, ranking as the second most effective design.
5. Dual minarets didn’t achieve the desired airspeed, but they are effective in distributing air within the mosque. Average air speeds of 1.6 ms^−1^ were recorded at the top of both minarets, which increased to 2.2 ms^−1^ in models with inclined roofs and sloping minarets.

**Table 2:** The average of physical measurements.

Case no	Position & number of minaret	Air velocity at the upper opening of the Minaret (ms^−1^)						
		Without an artificial air source with heating inside the minaret		Using an artificial air source with a speed of 1 m/s without heating inside the minaret		Using an artificial air source with a speed of 1 m/s with heating inside the minaret		Air temp. at the minaret top (^o^C)
The minaret has a regular square section & the mosque has a flat roof note: room temperature 26.5:28^o^								
a	In the middle of the Kiblah facade	> 0.4		1.7		1.9		47
c	In the west corner of the Kiblah facade	> 0.4		1.5		1.7		45
e	2 Minarets in Kiblah facade	0.4		1.4		1.6		45
g	In the middle of the Kiblah façade, the roof has a shokhshekha with openings	Minaret	0	Minaret	1	Minaret	1.4	50
		Shokhshikha	0	Shokhshikha	2.5	Shokhshikha	2.7	
	In the middle of the Kiblah façade, the roof has a shokhshekha without openings	0.4		2.3		2.5		50
The minaret has a sloped vertical section & the Mosque has an inclined roof Note: Room temperature 26.5:28^o^								
b	In the middle of the Kiblah facade	0.6		2.7		2.8		54
d	In the west corner of the Kiblah facade	0.6		2.00		2.5		52
f	2 Minarets in Kiblah facade	0.4		1.8		2.2		49
h	In the middle of the Kiblah facade, the roof has a shokhshekha without opening	0.8		3.00		3.2		57
i	In the Center of the Sloping pyramidal roof with sloping vertical section	1.00		3.8		5.00		60

### Computational results

**Figure 11:**
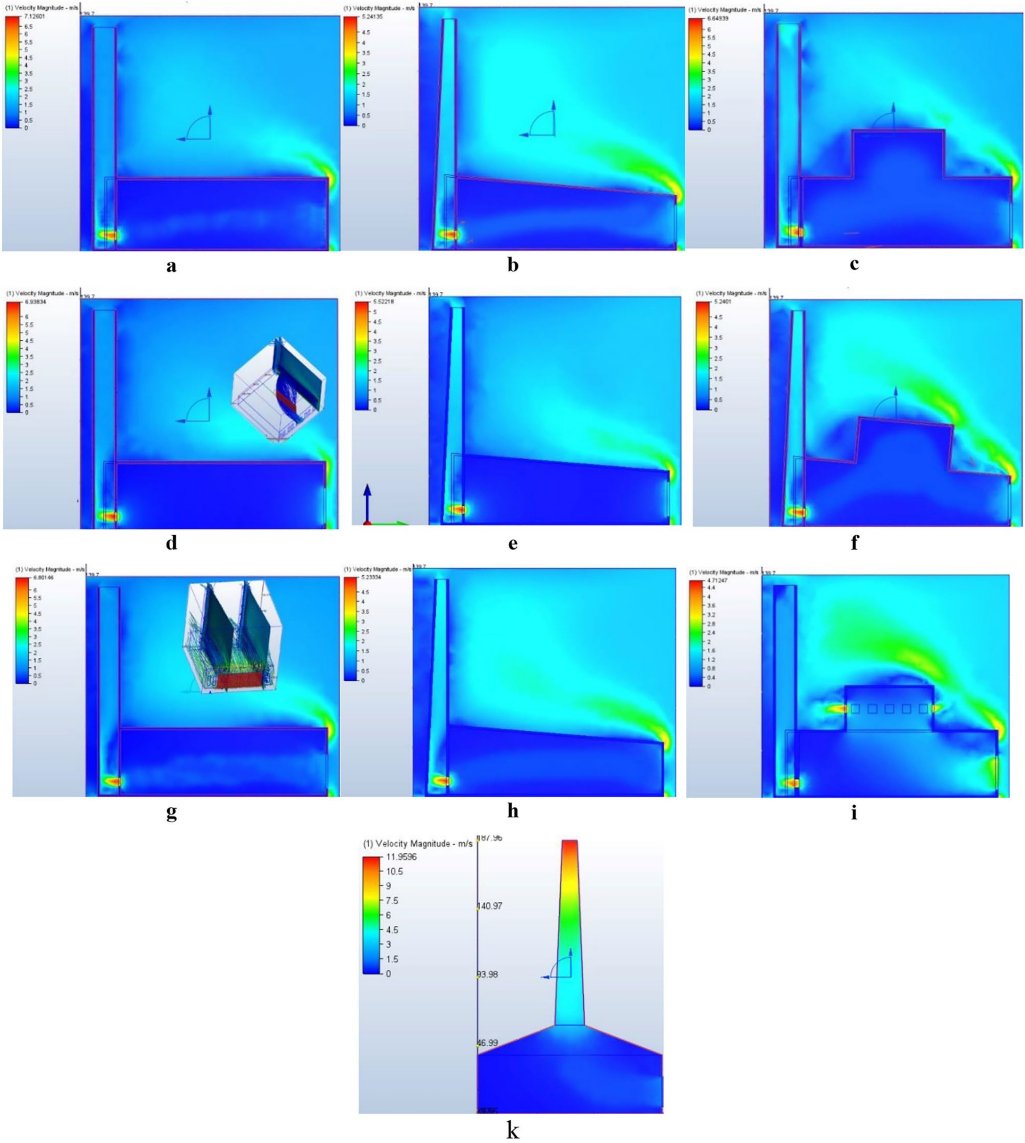
Studied cases CFD simulation results.

The computational analysis of the mosque models, conducted using Autodesk CFD software, the boundary conditions input data shown in Table 3. The CFD results closely mirrored the results pattern of the physical models with high accuracy as shown in Fig. 11, except the sloping minaret placed in the center of a pyramid roof the air velocity in the top of the CFD model was twice faster than air velocity of the physical measurement for the same model, but the air velocity is still the fastest among the models. This could be explained by construction material variation between the physical model and the CFD model. Again, this difference does not affect the experiment objective. The verification process showed good convergence between the experimental wind tunnel tests and the CFD simulations, demonstrating the model’s accuracy. However, CFD was superior to laboratory measurements in showing the distribution of air movement in the mosque space. It also showed that the peak air speed was at the bottom opening of the solar chimney, as the researcher measured the air speed in the laboratory at the top opening of the solar chimney, Due to the difficulty of measuring air movement in the physical model at the bottom opening of the solar chimney. The construction materials data input in the CFD model required the assistance of an expert to verify and revise the suitability of the materials for the simulation and reach the most accurate results possible. The key findings from the simulations were as follows:

1. Models with inclined roofs and sloping minarets consistently outperformed those with flat roofs and regular section minarets, aligning with the physical model observations.
2. In scenarios involving flat or inclined roofs, air velocity showed little variation, but there were notable differences in air distribution, as illustrated in Fig. 11a,b,d, e,g and h. The flat roof allows to be used for prayers, especially on hot summer nights, which makes the architect prefer it over the inclined roof, especially considering the small difference in air distribution.
3. To combine the advantages of a horizontal roof for prayers use and a inclined surface to improve air flow, the architect can make an interior suspended inclined ceiling
4. The inclusion of a shokhshekha with openings demonstrated excellent air distribution within the mosque, despite its adverse effect on the minaret’s function as a solar chimney. Conversely, a closed shokhshekha improved air velocity within the minaret and provided better air distribution compared to other scenarios, as seen in Fig. 11c,i and f. Architects should consider adding a shokhshekha or a dome for better air distribution flow.
5. The fastest air velocity design was found to be the sloping minaret placed in the center of a pyramid roof. The simulation results for this setup showed an air velocity of 11 ms^−1^ at the upper opening of the minaret, nearly double that of the physical models, confirming its effectiveness in both physical and computational models. For architects to effectively implement this model, they will need to thoroughly examine the structural system and construction materials used for building the minaret, as the traditional ones will make a punching for the roof that will add more structural elements to the plan which will restrict the space function.
6. The simulation also indicated that the most effective location for an air turbine is at the lower opening of the minaret, except in the case in Fig. 11k, where the upper opening was deemed more suitable.

**Table 3:** The boundary conditions CFD input data.

Wind velocity outside the model	Environment temperature	Inlet opening pressure	Outlet opening pressure	Wind direction	CFD used materials
1 ms^–1^	28°	1 Pa	0 Pa	north	Outside the model: air The model walls: bricks Minaret & mosque connected walls: heat insulation material

## Conclusion

The research explores the feasibility of transforming mosque minarets into solar chimneys, testing this hypothesis through a dual approach: laboratory experiments on 18 physical models representing 9 different cases, and computational simulations using Autodesk CFD for these cases. The findings from both experimental and computational methods revealed several key insights:

- Mosque minarets can be effectively converted into solar chimneys, enhancing natural ventilation and potentially generating energy for mosque operations through turbines installed at the minaret openings.
- The most efficient design for utilizing minarets as solar chimneys involves positioning a sloped minaret atop a pyramid roof. This configuration maximizes air velocity within the minaret, especially at its upper opening, making it an ideal spot for a wind turbine.
- Incorporating a shokhshekha with windows significantly improves air circulation within the mosque, although it somewhat hinders the minaret's effectiveness as a solar chimney.
- Sealing the shokhshekha windows or using closed glass improves the minaret’s performance as a solar chimney and enhances interior lighting.
- Models with inclined roofs and sloped minarets outperform those with flat roofs and regular cross-section minarets in terms of airspeed and distribution. Positioning the minaret in the middle of the Kiblah wall yields better results than corner placements. For corner installations, using two minarets, one at each corner, is advisable to optimize air distribution within the mosque space.

The author suggests future research to enhance the use of minarets as solar chimneys, focusing on varying minaret shapes (like circular or hexagonal), roof inclinations, and the number and placement of air entry openings. Exploring different mosque shapes and sizes, adjusting minaret dimensions, and varying the exposed surface area to solar radiation could also yield insights. Additionally, studying the impact of different construction materials to optimize temperature differences between mosques and minarets is recommended for further investigation. Installing a turbine fan inside the minaret to generate electricity would be a worthwhile addition to the investigation.

## Data Availability

All data used and/or analyzed during the current study are available from the corresponding author upon request.
